# Radiomic model versus R.E.N.A.L. nephrometry score for predicting the likelihood of undergoing partial nephrectomy in clear cell renal cell carcinoma

**DOI:** 10.3389/fruro.2026.1928982

**Published:** 2026-09-18

**Authors:** Fa-de Liu, Hui-li Yu, Du-jian Wang, Xiao-bing Ju, Yan-hao Yang

**Affiliations:** 1 Department of Urology, Jiangning Clinical Medical College of Kangda College of Nanjing Medical University, Nanjing, Jiangsu, China; 2 Department of Urology, The Affiliated Jiangning Hospital of Nanjing Medical University, Nanjing, Jiangsu, China; 3 Department of Urology, The First Affiliated Hospital of Nanjing Medical University, Nanjing, Jiangsu, China

**Keywords:** carcinoma, machine learning, nephrectomy, nephrometry score, radiomics, renal cell, tomography

## Abstract

**Objectives:**

To develop a CT-based radiomic model for predicting partial nephrectomy (PN) in clear cell renal cell carcinoma (ccRCC) and compare it with the R.E.N.A.L. Nephrometry Score (RNS).

**Methods:**

This retrospective study included 413 ccRCC patients who underwent PN or radical nephrectomy. The RNS was calculated from preoperative CT. Radiomic features were extracted from corticomedullary-phase CT. Reproducibility was assessed using intraclass correlation coefficients (ICCs). LASSO regression was used for feature selection and Rad-score construction. Multivariable logistic regression models were built based on the total RNS sum and the Rad-score. Performance was evaluated by AUC, calibration, and decision curve analysis.

**Results:**

Of 1210 extracted features (after excluding general_info fields), 851 showed good to excellent reproducibility (ICC > 0.75). The radiomic model (6 selected features) achieved an AUC of 0.886 (95% CI: 0.81-0.91) in the internal validation cohort, significantly outperforming the RNS model (AUC = 0.793, 95% CI: 0.72-0.85; DeLong test, P = 0.015). The radiomic model also demonstrated better calibration (Brier score: 0.11 vs. 0.16) and higher clinical net benefit. SHAP analysis identified tumor size and texture heterogeneity as the strongest predictors.

**Conclusion:**

A CT-based radiomic model provides superior accuracy over the RNS for preoperatively predicting PN likelihood in ccRCC and holds significant potential to augment surgical decision-making and personalize treatment planning. Prospective multi-center validation is warranted before routine clinical adoption.

## Introduction

Renal cell carcinoma (RCC) is the seventh most common malignancy globally, with the highest mortality rate among urological cancers (1). Clear cell RCC (ccRCC), accounting for 70-80% of RCC cases, is the most prevalent histological subtype (1). Surgical resection remains the cornerstone of curative treatment for localized ccRCC, with partial nephrectomy (PN) and radical nephrectomy (RN) as primary options (2). PN is the standard of care for localized renal masses, providing oncological outcomes comparable to RN while preserving renal function and reducing the risk of chronic kidney disease (2, 3).

Over ten scoring systems, including the R.E.N.A.L. nephrometry score (RNS), PADUA score, and C-index, have been proposed to quantify the anatomical complexity of renal tumors (4, 5). Among these, the RNS, developed by Kutikov and Uzzo (5), is the most widely used standardized system. It classifies tumors into low (4-6), moderate (7-9), and high (10-12) complexity groups based on cross-sectional imaging, which correlate with surgical difficulty and the likelihood of PN (5, 6). We chose RNS over other scoring systems for several reasons: (1) its widespread clinical adoption and ease of calculation from routine CT; (2) its well-established correlation with perioperative outcomes and PN success rates; (3) its extensive validation across multiple centers, facilitating comparison with existing literature; and (4) the intuitive, component-based structure that allows direct comparison with radiomic features. However, while RNS provides a standardized assessment of anatomical complexity, it offers limited insight into the underlying tumor biology (7).

Preoperative biopsy can assess tumor grade and biology but is invasive, prone to sampling errors, and carries risks (8–10). A non-invasive tool capable of providing quantitative assessment of tumor characteristics beyond conventional morphology—including features that may reflect underlying tissue heterogeneity—is urgently needed. Radiomics, the high-throughput extraction of quantitative features from medical images, addresses this gap by transforming images into actionable data (11, 12). Subvisual textures have been shown to correlate with pathological grades, genotypes, and clinical outcomes across various cancers, including ccRCC (13, 14). Recent radiomics studies have demonstrated the ability to characterize renal tumor heterogeneity and correlate with pathological features such as Fuhrman grade (15–17). More relevant to the present study, emerging evidence suggests that CT-based radiomic features may also capture information relevant to surgical complexity and perioperative outcomes (3, 6).

While RNS predicts surgical complexity, the ultimate clinical question is often binary: “Is this patient likely to undergo PN?” We hypothesized that a radiomic model, which captures quantitative imaging features beyond conventional morphological assessment, may provide additional discriminatory information for predicting the actual surgical decision regarding PN, compared to the RNS alone. Notably, this endpoint reflects institutional surgical decision-making patterns, which integrate tumor anatomy, surgeon expertise, patient comorbidities, and institutional preferences, rather than an objective measure of tumor resectability. Thus, this study aimed to: 1) develop a robust radiomic model to predict the likelihood of undergoing PN; 2) construct a predictive model based on key RNS components; 3) directly compare the performance of these two approaches; 4) ensure methodological robustness through rigorous feature reproducibility analysis; and 5) enhance model interpretability through SHAP-based explanation of the final model’s predictions.

## Materials and methods

### Study population and surgical decision context

A consecutive cohort of 413 patients who underwent PN or RN for pathologically confirmed ccRCC between June 2019 and April 2024 was identified from our institutional surgical pathology database. A flow diagram of patient selection is provided in **Supplementary Figure 1**. Of 482 initially identified patients with surgically treated renal masses during the study period, 69 were excluded: 24 for absence of three-phase CT, 18 for significant motion artifacts, 15 for tumors with severe hemorrhage/necrosis precluding segmentation, 7 for prior RCC treatment, and 5 for distant metastasis at diagnosis, leaving 413 patients for final analysis. Inclusion criteria were: (1) pathologically confirmed ccRCC; (2) availability of preoperative three-phase contrast-enhanced CT scans performed within 3 months prior to surgery; (3) complete clinical and pathological records. Exclusion criteria were: (1) tumors with severe hemorrhage or necrosis preventing accurate segmentation; (2) images with significant motion artifacts; (3) absence of three-phase CT images; (4) prior treatment for RCC (e.g., ablation, targeted therapy, radiotherapy); (5) presence of distant metastasis at diagnosis.

The endpoint for model development was the final surgical procedure performed, as documented in the operative records. In cases where a planned PN was converted to RN intraoperatively (n = 4, 1.0% of the cohort), the final procedure (RN) was used as the endpoint. These conversions were retained in the RN group for analysis. Patients with imperative indications for nephron-sparing surgery—including solitary kidney (n = 8), bilateral or multifocal tumors (n = 12), hereditary syndromes (n = 3), and preexisting renal dysfunction (eGFR < 60 mL/min/1.73m², n = 31)—were included in the cohort. These factors were not incorporated as covariates in the primary models, as they represent independent clinical indications for PN that are not captured by either RNS or radiomics; however, they were balanced between the PN and RN groups (**Supplementary Table 1**).

Surgical decision-making process: The choice between PN and RN was made by attending urologic surgeons based on a combination of tumor anatomical characteristics (assessed by imaging), patient-specific factors (including age, comorbidities, baseline estimated glomerular filtration rate), and surgeon expertise. No strict algorithmic criteria were applied, reflecting real-world clinical practice. The RNS was calculated retrospectively for this study and was not used by treating surgeons in their preoperative decision-making. Thus, our model predicts the institutional decision outcome rather than an objective measure of tumor resectability.

This single-institution retrospective study was approved by the Institutional Review Board of the First Affiliated Hospital of Nanjing Medical University (approval number: 202400528), and the requirement for written informed consent was waived due to the retrospective nature of the study. All procedures were performed in accordance with the 1964 Helsinki Declaration and its later amendments.

### CT image acquisition

All patients underwent abdominal CT using a 64-section multidetector CT scanner (SOMATOM Definition AS+, Siemens Healthineers, Forchheim, Germany), with standardized parameters used in daily clinical practice. CT imaging parameters included: tube voltage of 120 kVp, effective tube current of 180 mAs, rotation time of 0.5 s, detector collimation of 32 × 1.2 mm, field of view of 350 × 350 mm, matrix of 512 × 512, and reconstruction section thickness of 1.5 mm. After unenhanced scanning, an intravenous bolus of non-ionic contrast material (iopromide, Ultravist 300; Bayer Schering Pharma, Berlin, Germany) was administered at a dose of 1.5 mL/kg body weight (maximum 120 mL), injected at a rate of 3.0 mL/s via a power injector through antecubital venous access. Arterial phase scanning was triggered automatically using bolus-tracking software (Care-Bolus; Siemens Medical Systems, Iselin, NJ), with a threshold of 100 HU at the level of the supraceliac abdominal aorta, beginning 10 s after threshold attainment. Corticomedullary phase scanning was acquired with a fixed 40-second delay after arterial phase completion. The corticomedullary phase was selected for radiomic analysis based on previous studies indicating that this phase best captures tumor vascularity and heterogeneity, which are key correlates of tumor biology (16, 18). While nephrographic phase images may provide additional insights, prior research has shown that single-phase corticomedullary analysis achieves comparable or superior performance for tumor characterization compared to multiphasic analysis (19). This approach also facilitates clinical translation and reduces computational burden.

### Image preprocessing

Prior to feature extraction, all CT images underwent standardized preprocessing to minimize batch effects and ensure feature reproducibility in accordance with the Image Biomarker Standardization Initiative (IBSI) guidelines (20). Images were resampled to isotropic voxel spacing of 1 × 1 × 1 mm³ using third-order B-spline interpolation to maintain spatial consistency across patients. Normalization was performed by remapping the histogram to fit within μ ± 3σ, where μ represents the mean gray-level intensity within the volume of interest (VOI) and σ is the standard deviation. This method minimizes variations due to differences in contrast administration and scanner calibration. Gray-level discretization was applied with a fixed bin width of 64, selected based on a sensitivity analysis of 50 cases, where bin widths of 16, 25, 32, and 64 were evaluated. A bin width of 64 provided the optimal balance between feature reproducibility (intraclass correlation coefficient [ICC] > 0.75) and preservation of texture information, consistent with previous renal radiomics studies (21). Laplacian of Gaussian (LoG) filtering was applied with sigma values of 2.0, 3.0, and 4.0 mm to extract fine, medium, and coarse texture patterns, respectively. Wavelet transform was performed using the Daubechies 4 (db4) wavelet basis function via the PyWavelet package (version 0.5.2). The db4 wavelet was selected for its orthogonality, compact support, and suitability for medical image analysis. Single-level decomposition generated eight subband images per original image, capturing frequency-based texture information across three-dimensional orientations. A single urologist (Y.H.Y.), blinded to the radiomic analysis and pathological results, calculated the RNS for each tumor based on the preoperative CT scans (5). The RNS was calculated retrospectively for this study; treating surgeons did not use the RNS formally in their preoperative decision-making. The total RNS sum and individual components (R, E, N, A, L) were recorded for all patients. Tumors were classified as low (4-6), moderate (7-9), or high (10-12) complexity based on the total score.

Two radiologists (Reader 1 with 8 years of experience; Reader 2 with 10 years of experience) and one urological surgeon (Reader 3 with 5 years of experience), all blinded to clinical and pathological details, performed manual tumor segmentation (**Figure 1**). Regions of interest (ROIs) were delineated along the tumor contour on each transverse section using an open-source imaging platform (ITK-SNAP, version 3.6.0; www.itksnap.org), carefully avoiding adjacent normal renal parenchyma, perinephric fat, and macroscopic calcifications. ROIs were initially delineated on the corticomedullary phase and eventually rendered in three dimensions as VOIs. To assess inter-observer reproducibility, all three readers independently performed tumor segmentation on 50 randomly selected cases (approximately 12% of the cohort, stratified by tumor size and complexity). The 50 cases used for reproducibility analysis were randomly selected from the entire cohort prior to train-validation split, stratified by tumor size and complexity. All preprocessing parameters (including bin width selection) were optimized using these 50 cases only, and the final parameter settings were then applied to the full dataset before splitting. To assess intra-observer reproducibility, Reader 1 repeated the segmentation on the same 50 cases after a two-week interval. ICCs were calculated using a two-way random-effects model for absolute agreement (ICC(2,1), as defined by Shrout and Fleiss), the recommended approach for radiomics reproducibility studies per IBSI guidelines (20). Features with ICC > 0.75 for both inter- and intra-observer agreement were considered to have good to excellent reproducibility and were retained for further analysis (22). For the remaining 363 cases, segmentation was performed by Reader 1, with random quality checks by Reader 2.

**Figure 1:**
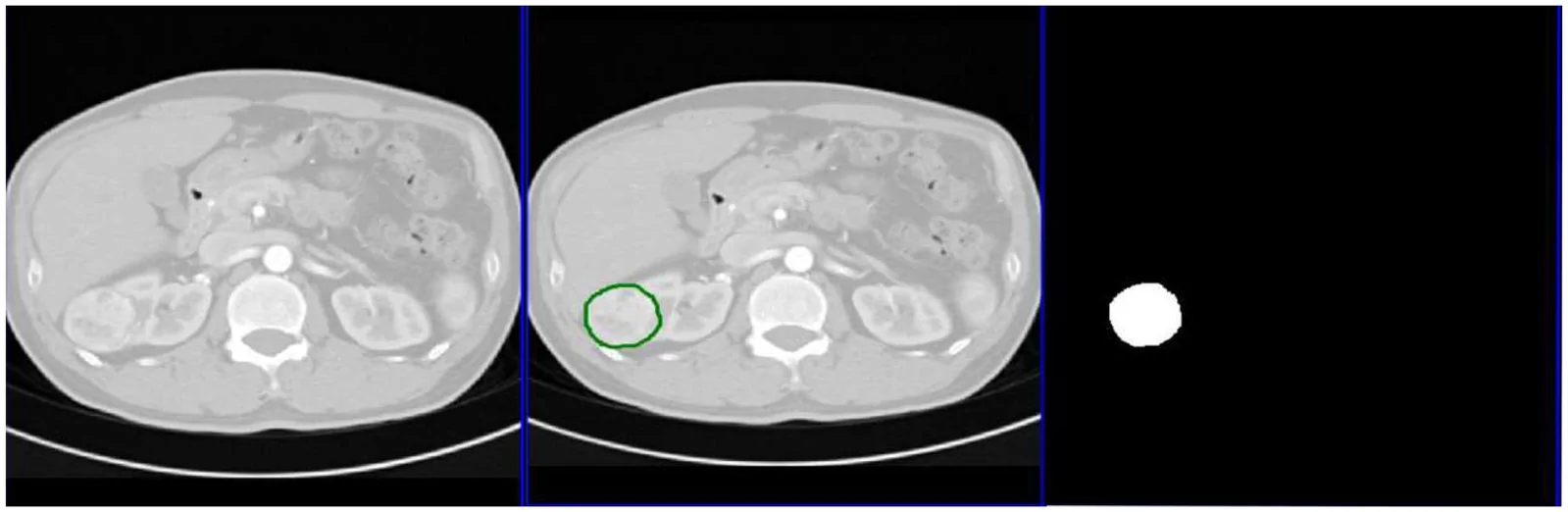
Tumors are segmented manually on corticomedullary phase CT sections. Axial image showing tumor delineation (green contour) and 3D rendering of the segmented tumor volume.

Radiomic feature extraction was conducted using PyRadiomics (version 3.0.1; https://pyradiomics.readthedocs.io/) within Python (version 3.7.4). A total of 1210 features were extracted from each VOI, covering seven feature classes: 1. First-order statistics (n = 18), characterizing voxel intensity distributions. 2. Shape-based features (3D) (n = 14), describing the tumor’s three-dimensional size and morphology. 3. Gray-Level Co-occurrence Matrix (GLCM) (n = 24), quantifying texture patterns based on pixel pair distributions. 4. Gray-Level Run Length Matrix (GLRLM) (n = 16), capturing runs of consecutive pixels with identical gray-level values. 5. Gray-Level Size Zone Matrix (GLSZM) (n = 16), quantifying zones of connected voxels sharing the same gray-level value. 6. Neighboring Gray Tone Difference Matrix (NGTDM) (n = 5), quantifying differences between a voxel’s gray value and the average of its neighbors. 7. Gray-Level Dependence Matrix (GLDM) (n = 14), quantifying dependencies between adjacent voxels with similar gray levels. All `general_info` fields were excluded from the feature matrix prior to modeling to avoid segmentation-derived metadata. All features were named following PyRadiomics conventions and IBSI guidelines (20).

### Feature selection and model development

The dataset was randomly divided into a training cohort (70%, n = 289) and an internal validation cohort (30%, n = 124) using stratified sampling based on surgical outcome (PN vs. RN) to ensure balanced distribution. The train-validation split was performed at the patient level; all patients in this cohort had a single tumor. Feature selection and model development were exclusively conducted within the training cohort to avoid data leakage. Radiomic features were standardized through Z-score normalization before applying LASSO regression. All 851 reproducible features (excluding general_info fields) were entered directly into the LASSO regression without prior correlation-based filtering. LASSO logistic regression was used to identify the most predictive features for PN likelihood. LASSO, a regularization technique, adds a penalty term to the logistic regression cost function, shrinking the coefficients of less important features toward zero and effectively performing feature selection (23). The optimal tuning parameter was determined through 10-fold cross-validation using binomial deviance as the loss function, repeated 10 times with different random folds to ensure stability. The λ value that minimized the cross-validated deviance (λ_min) was selected. Features with non-zero coefficients at λ_min were retained for the final model. The stability of feature selection across the 10 repeats of 10-fold cross-validation was assessed: the selected features were chosen in 9 of 10 repeats, indicating good selection stability. The selected features were combined linearly using their standardized LASSO coefficients to compute a radiomic score (Rad-score) for each patient. To prevent overfitting, a multi-layered strategy was implemented: (1) feature selection was performed solely within the training cohort; (2) LASSO regularization inherently controls overfitting by shrinking coefficients; (3) model performance was assessed exclusively in the internal validation cohort; (4) 1000 bootstrap resamples were used to calculate 95% confidence intervals for all performance metrics, providing robust uncertainty estimates; (5) the events per variable (EPV) ratio in the training cohort exceeded 10 for all models, meeting standard criteria for logistic regression.

### Model construction

Two predictive models were developed using multivariable logistic regression in the training cohort:

1. RNS Model: The total RNS sum (as a continuous variable) was used as the predictor to predict the likelihood of PN.
2. Radiomic Model: The Rad-score from the LASSO-selected features was used as the predictor.

Additionally, a combined model integrating the Rad-score with total RNS sum was explored to assess potential incremental value. The ‘A’ (anterior/posterior) component was excluded from the RNS model as it does not contribute to surgical complexity in the same manner as the other components and is not consistently associated with PN likelihood in prior studies (5).

### Statistical analysis

Categorical variables were compared using the χ² test or Fisher’s exact test, as appropriate. Continuous variables were tested for normality with the Shapiro-Wilk test. Normally distributed continuous variables were compared using the Student’s t-test, while non-normally distributed variables (including BMI) were compared using the Mann-Whitney U test. All tests were conducted with SPSS software (version 20.0; IBM Corp., Armonk, NY, USA). Given the multiple comparisons (k = 7 clinical variables in **Table 1**), a Bonferroni-corrected significance level of α = 0.05/7 ≈ 0.007 was applied to control for Type I error. Statistical analyses were performed with R software (version 3.5.2; R Foundation for Statistical Computing, Vienna, Austria), using the following packages: glmnet for LASSO regression, pROC for ROC analysis, rmda for decision curve analysis (DCA), and shapr for SHAP analysis. The performance of all models was evaluated in the internal validation cohort using multiple metrics: (1) Discrimination: Assessed by the area under the receiver operating characteristic curve (AUC), with 95% CIs calculated using 1000 bootstrap resamples; (2) Calibration: Assessed using calibration plots and the Brier score, which measures the mean squared difference between predicted probabilities and actual outcomes (lower scores indicate better calibration). The Hosmer-Lemeshow goodness-of-fit test was also conducted; (3) Clinical utility: Assessed using DCA, which quantifies the net benefit of using the model across a range of threshold probabilities (24). The DeLong test was used to compare AUCs between models, with the Z-statistic reported alongside the P-value. A two-sided P-value < 0.05 was considered statistically significant. To enhance interpretability, SHAP (SHapley Additive exPlanations) analysis was conducted on the internal validation cohort (25) (Lundberg & Lee, 2017). SHAP analysis was performed on the final logistic regression model that used the Rad-score as the sole predictor (the ‘Radiomic Model’). To derive feature-level SHAP values, we first computed the Rad-score for each patient as the linear combination of the selected radiomic features weighted by their LASSO coefficients. SHAP values were then calculated for each of these features using the KernelExplainer implementation (25), with the logistic regression model’s predicted probability as the output. SHAP values quantify the contribution of each feature to the model’s prediction for individual patients, with positive values indicating an increased likelihood of PN and negative values indicating a decreased likelihood. The magnitude of SHAP values reflects the strength of each feature’s contribution.

**Table 1:** Clinical and pathological characteristics of patients.

Characteristic	Partial nephrectomy (n=312)	Radical nephrectomy (n=101)	P-value*
Age (years), mean ± SD	55.69 ± 13.33	57.69 ± 11.71	0.177
Gender			0.032
Male	200 (64.1%)	76 (75.2%)	
Female	112 (35.9%)	25 (24.8%)	
BMI (kg/m²), median (IQR)	22.0 (19.8, 25.1)	23.0 (18.5, 25.2)	0.82^†^
Hypertension	195 (62.5%)	50 (49.5%)	0.55
Diabetes mellitus	149 (47.8%)	27 (26.7%)	0.16
Smoking	136 (43.6%)	24 (23.8%)	0.17
Side			0.82
Right	160 (51.3%)	49 (48.5%)	
Left	152 (48.7%)	52 (51.5%)	
Fuhrman grade			<0.001
I or II (low-grade)	205 (65.7%)	38 (37.6%)	
III or IV (high-grade)	107 (34.3%)	63 (62.4%)	
R.E.N.A.L. Nephrometry Score			<0.001
Low complexity (4-6)	145 (46.5%)	26 (25.7%)	
Moderate complexity (7-9)	128 (41.0%)	68 (67.3%)	
High complexity (10-12)	39 (12.5%)	7 (6.9%)	

Data are presented as n (%) unless otherwise indicated. SD, standard deviation; IQR, interquartile range. *P-values from χ² test for categorical variables, t-test for age. Bonferroni-corrected significance threshold α = 0.007. ^†^Mann-Whitney U test (BMI non-normally distributed by Shapiro-Wilk test, P < 0.05).

## Results

### Patient characteristics

The clinical and pathological characteristics of the 413 patients are summarized in **Table 1**. Among the cohort, 312 patients (75.5%) underwent PN, while 101 patients (24.5%) underwent RN. The association between RNS complexity and RN rate was non-monotonic: 15.2% for low-complexity tumors (RNS 4-6), 34.7% for moderate-complexity tumors (RNS 7-9), and 15.2% for high-complexity tumors (RNS 10-12) (P < 0.001 by chi-square test for overall association across the three complexity groups) (**Table 1**). The highest RN rate was observed in the moderate-complexity group rather than the high-complexity group. The lower RN rate in the high-complexity group (15.2%, based on 7 RN events among 46 patients; 95% CI for this proportion: 6.4-28.9%) compared to the moderate-complexity group (34.7%) is noteworthy. Several explanations are possible, including selection bias, the small number of events in this subgroup, or institutional practice patterns favoring attempted PN even in complex cases. This pattern should be interpreted cautiously.

### Feature reproducibility and selection

Of the 1210 extracted radiomic features (after excluding general_info fields), 851 (70.3%) demonstrated good to excellent reproducibility, with an ICC > 0.75 for both inter- and intra-observer agreement, and were retained for further analysis. The median ICC for retained features was 0.89 (IQR: 0.82-0.94), indicating robust segmentation consistency. LASSO regression identified six robust radiomic features predictive of the likelihood of PN (λ_min = 0.0626, log(λ) ≈ -2.77) (**Figure 2**, **Table 2**). The selected features included: original_shape_Maximum2DDiameterSlice, original_shape_Maximum3DDiameter, log-sigma-2-0-mm-3D_firstorder_Energy, log-sigma-2-0-mm-3D_glcm_Imc1, log-sigma-2-0-mm-3D_ngtdm_Strength, and log-sigma-3-0-mm-3D_gldm_LargeDependenceHighGrayLevelEmphasis. Notably, tumor volume-related features were selected, consistent with the well-established correlation between tumor size and surgical complexity (26). Texture features from GLCM, NGTDM, and GLDM classes were also selected, suggesting that intratumoral heterogeneity captured by these features may reflect biological factors influencing surgical resectability.

**Figure 2:**
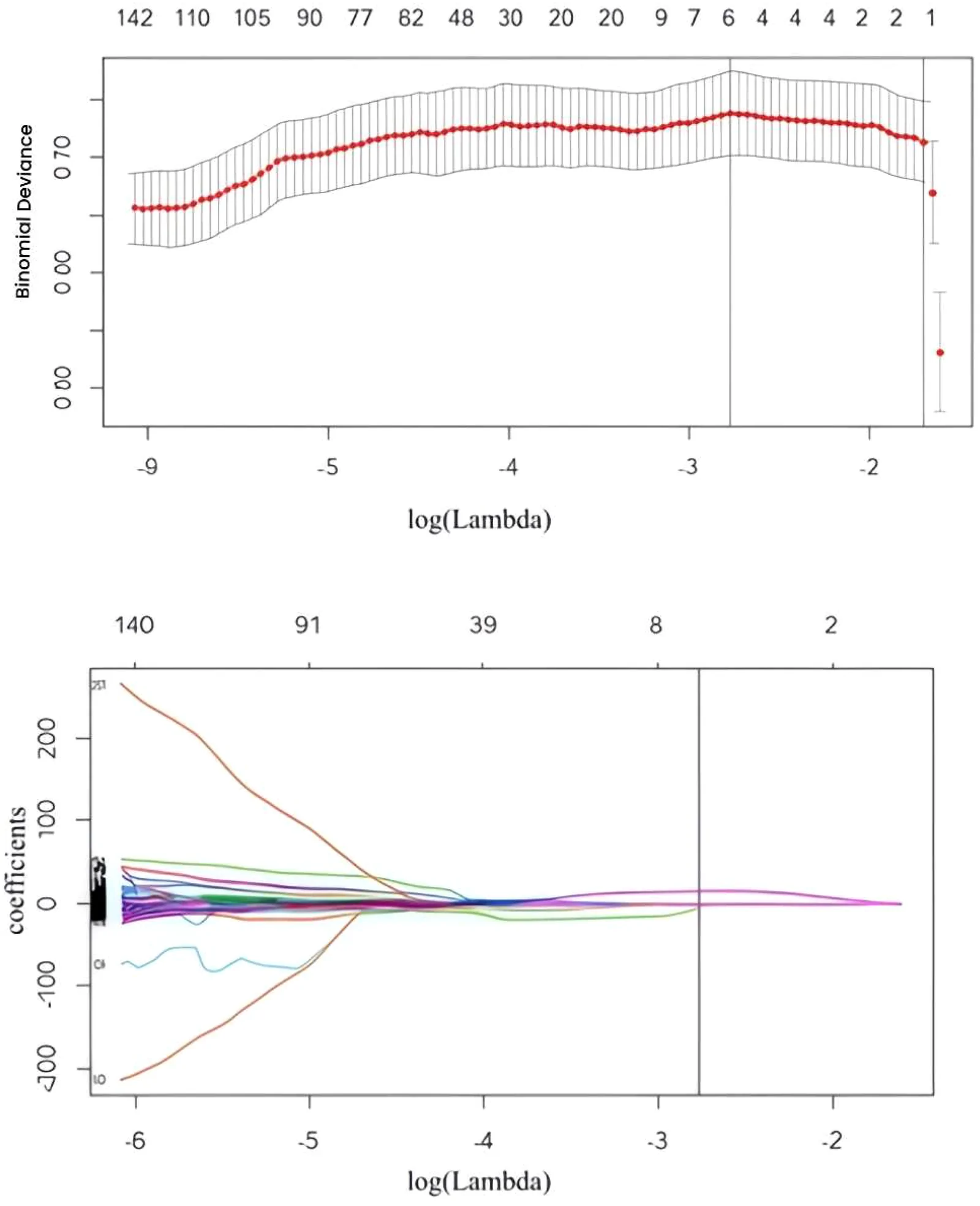
Radiomic feature selection using LASSO logistic regression. Selection of tuning parameter (l) using 10-fold cross-validation with binomial deviance as the loss function. The x-axis shows log(l) from -8 to 0, and the y-axis shows cross-validated deviance. The gray shaded area represents the 95% confidence interval of the deviance. The optimal l value of 0.0626 (log(l) ≈ -2.77) was chosen based on minimum deviance criteria (left vertical line). LASSO coefficient profiles of the 851 reproducible features across log(l) values. Each colored line represents a feature coefficient. The vertical line at optimal l selects six features with non-zero coefficients.

**Table 2:** Radiomic features selected by LASSO regression analysis.

Radiomic feature	Standardized coefficient	P-value	Feature class	ICC (Intra-observer)	ICC (Inter-observer)
original_shape_Maximum2DDiameterSlice	0.992	0.088	Shape	0.97	0.95
original_shape_Maximum3DDiameter	-0.096	0.873	Shape	0.92	0.89
log.sigma.2.0.mm.3D_glcm_Imc1	-0.135	0.357	GLCM	0.95	0.92
log.sigma.2.0.mm.3D_ngtdm_Strength	-0.075	0.700	NGTDM	0.94	0.91
log.sigma.3.0.mm.3D_gldm_LargeDependenceHighGrayLevelEmphasis	0.074	0.659	GLDM	0.96	0.93
log.sigma.2.0.mm.3D_firstorder_Energy	-0.071	0.784	First-order	0.91	0.88

Coefficients are standardized to allow direct comparison of feature importance. GLCM, Gray-Level Co-occurrence Matrix; GLDM, Gray-Level Dependence Matrix; NGTDM, Neighboring Gray Tone Difference Matrix; ICC, intraclass correlation coefficient (two-way random-effects model for absolute agreement). All ICC > 0.75 indicate good to excellent reproducibility. P-values are from a multivariable logistic regression model fit using the six selected features as simultaneous predictors; non-significant individual P-values are expected due to LASSO’s joint selection and multicollinearity among radiomic features.

The P-values reported in **Table 2** are derived from a multivariable logistic regression model fit using the six selected features as simultaneous predictors. The non-significant individual P-values (range: 0.088 to 0.873) are not unexpected in the context of LASSO-selected features, as LASSO selects features based on their joint predictive contribution rather than individual marginal significance, and multicollinearity among correlated radiomic features can inflate standard errors and mask individual significance despite strong joint predictive performance. The strong joint predictive performance of the Rad-score (AUC = 0.886 in the internal validation cohort) confirms that these features collectively contribute meaningful predictive information.

### Model performance

RNS model: In the internal validation cohort, the RNS model, using the total RNS sum, achieved an AUC of 0.793 (95% CI: 0.72-0.85) for predicting the likelihood of PN.

Radiomic model: The radiomic model, using the Rad-score alone, demonstrated significantly superior discrimination, with an AUC of 0.886 (95% CI: 0.81-0.91) (DeLong test, Z = 2.43, P = 0.015) (**Figure 3**, **Table 3**). The radiomic model exhibited better sensitivity (83.9% vs. 71.3%), specificity (82.1% vs. 74.6%), and accuracy (83.2% vs. 72.8%) using the Youden-index optimal threshold.

**Figure 3:**
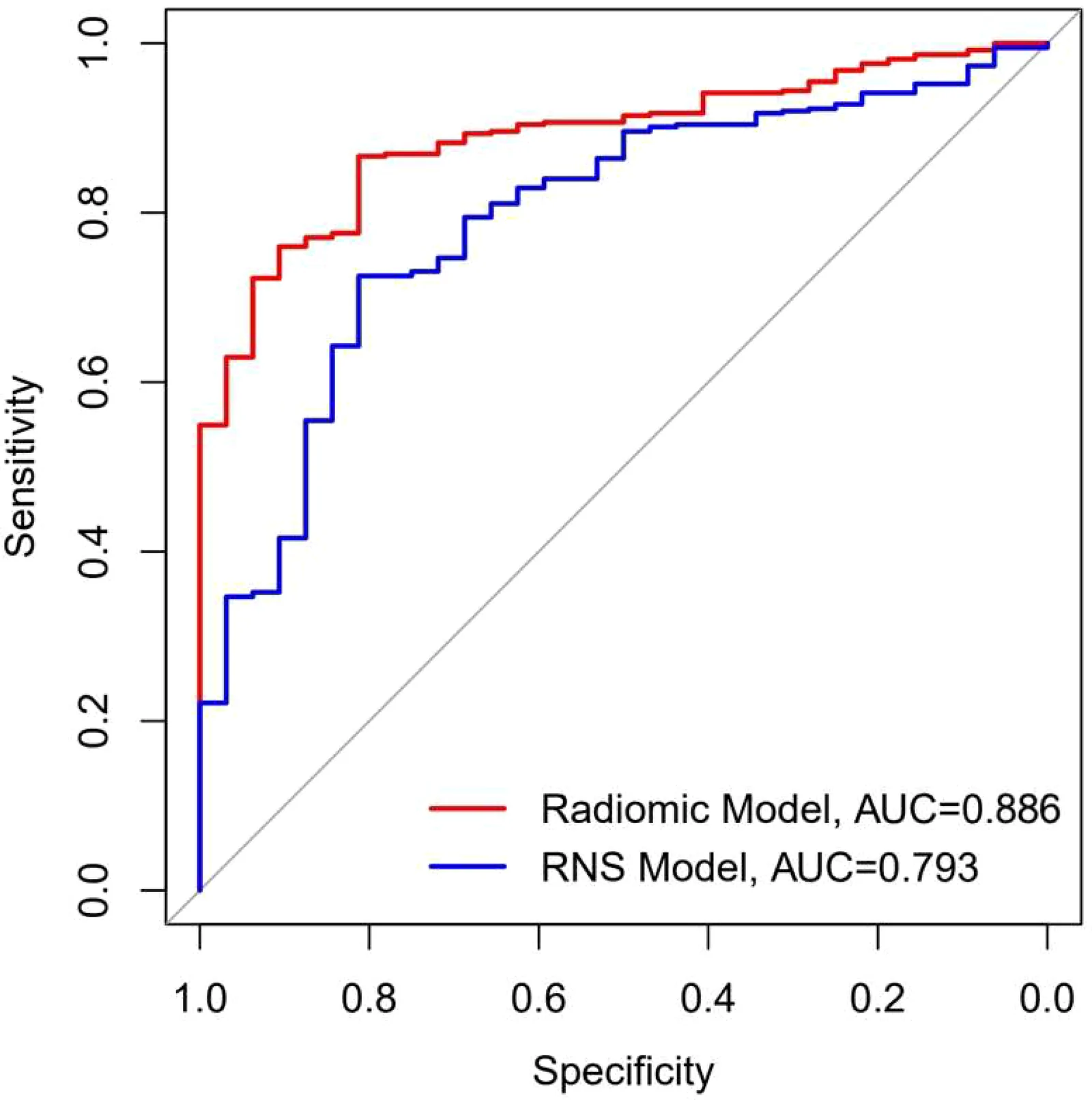
Comparison of model performance. Receiver operating characteristic (ROC) curves for the R.E.N.A.L. Nephrometry Score (RNS) model and the Radiomic model in predicting the likelihood of partial nephrectomy (PN) in the internal validation cohort.

**Table 3:** Performance metrics of models in validation cohort.

Model	AUC (95% CI)	Sensitivity (%)	Specificity (%)	PPV (%)	NPV (%)	Accuracy (%)	Brier score
RNS Model	0.793 (0.72-0.85)	71.3	74.6	69.8	76.2	72.8	0.16
Radiomic Model	0.886 (0.81-0.91)	83.9	82.1	81.7	85.0	83.2	0.11
Combined Model	0.888 (0.83-0.94)	83.9	83.1	82.0	85.1	83.6	0.11

*RNS, R.E.N.A.L. Nephrometry Score; AUC, area under the curve; CI, confidence interval (calculated using 1000 bootstrap resamples); PPV, positive predictive value; NPV, negative predictive value. Combined model includes Rad-score + total RNS sum. PPV and NPV are calculated based on the internal validation cohort prevalence (PN: 75.5%, RN: 24.5%). Sensitivity, specificity, PPV, NPV, and accuracy are reported at the Youden-index optimal threshold.

Combined model: The combined model integrating Rad-score with total RNS sum achieved an AUC of 0.888 (95% CI: 0.83-0.94), which was not significantly different from the radiomic model alone (P = 0.62).

Calibration curves showed good agreement between predicted probabilities and observed outcomes for both models, though the radiomic model had a closer fit to the ideal calibration line (**Figure 4**). The Brier score was lower for the radiomic model (0.11) compared to the RNS model (0.16), indicating better overall probabilistic prediction accuracy (which encompasses both calibration and discrimination). The Hosmer-Lemeshow test was non-significant for both models (radiomic: P = 0.43; RNS: P = 0.28), which is consistent with adequate calibration but should not be interpreted as definitive evidence of perfect calibration. DCA demonstrated that the radiomic model provided a greater net benefit across most clinically relevant threshold probabilities compared to the RNS model and the default strategies of treating all or no patients with PN (**Figure 5**). At a threshold probability of 0.6, the radiomic model yielded a net benefit of 0.32 compared to 0.21 for the RNS model. The threshold of 0.6 was selected based on clinical considerations (representing a clinically meaningful probability threshold at which most surgeons would consider PN planning) and was visually identified as the region where the radiomic model’s net benefit curve showed the greatest separation from the RNS model’s curve. We acknowledge this is an exploratory threshold and should not be interpreted as an objectively ‘optimal’ threshold derived from formal optimization.

**Figure 4:**
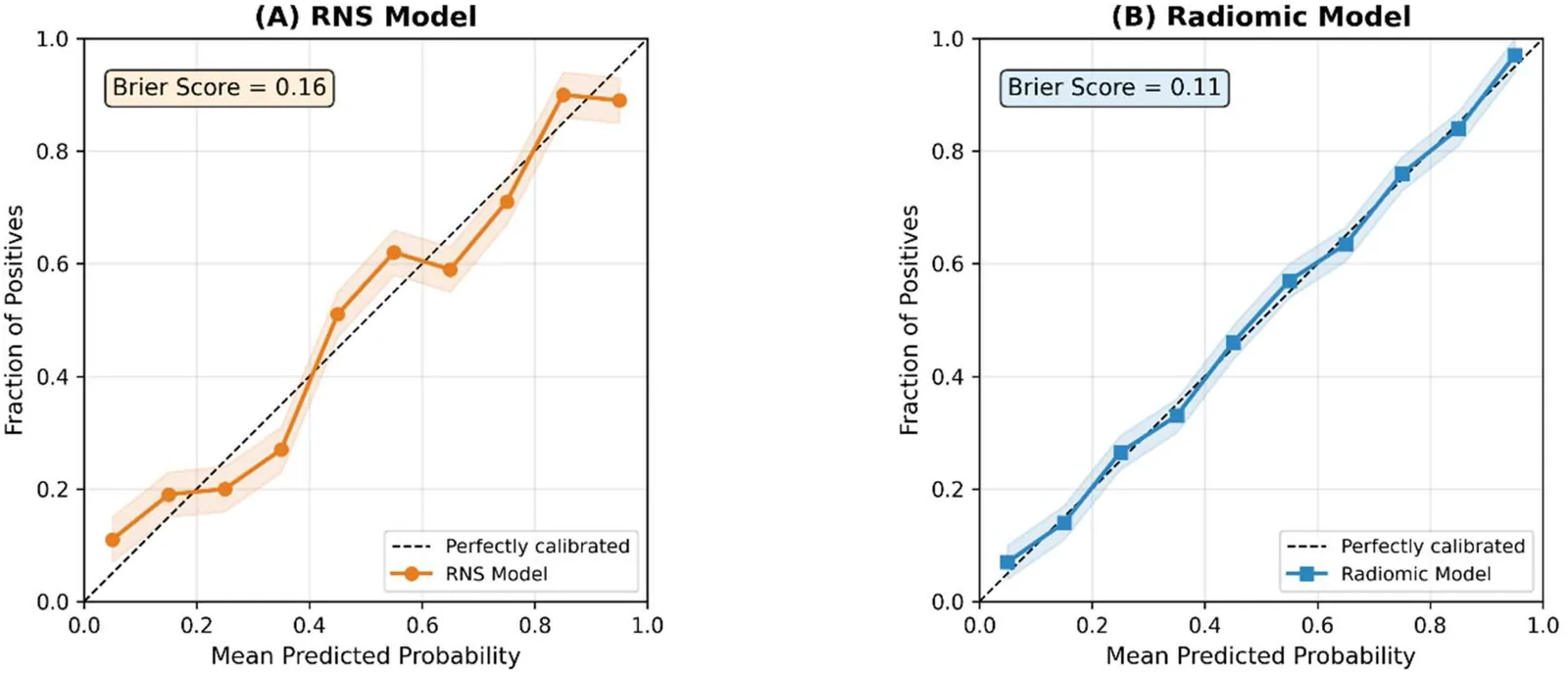
Calibration curves for the RNS model **(A)** and Radiomic model **(B)** in the internal validation cohort.

**Figure 5:**
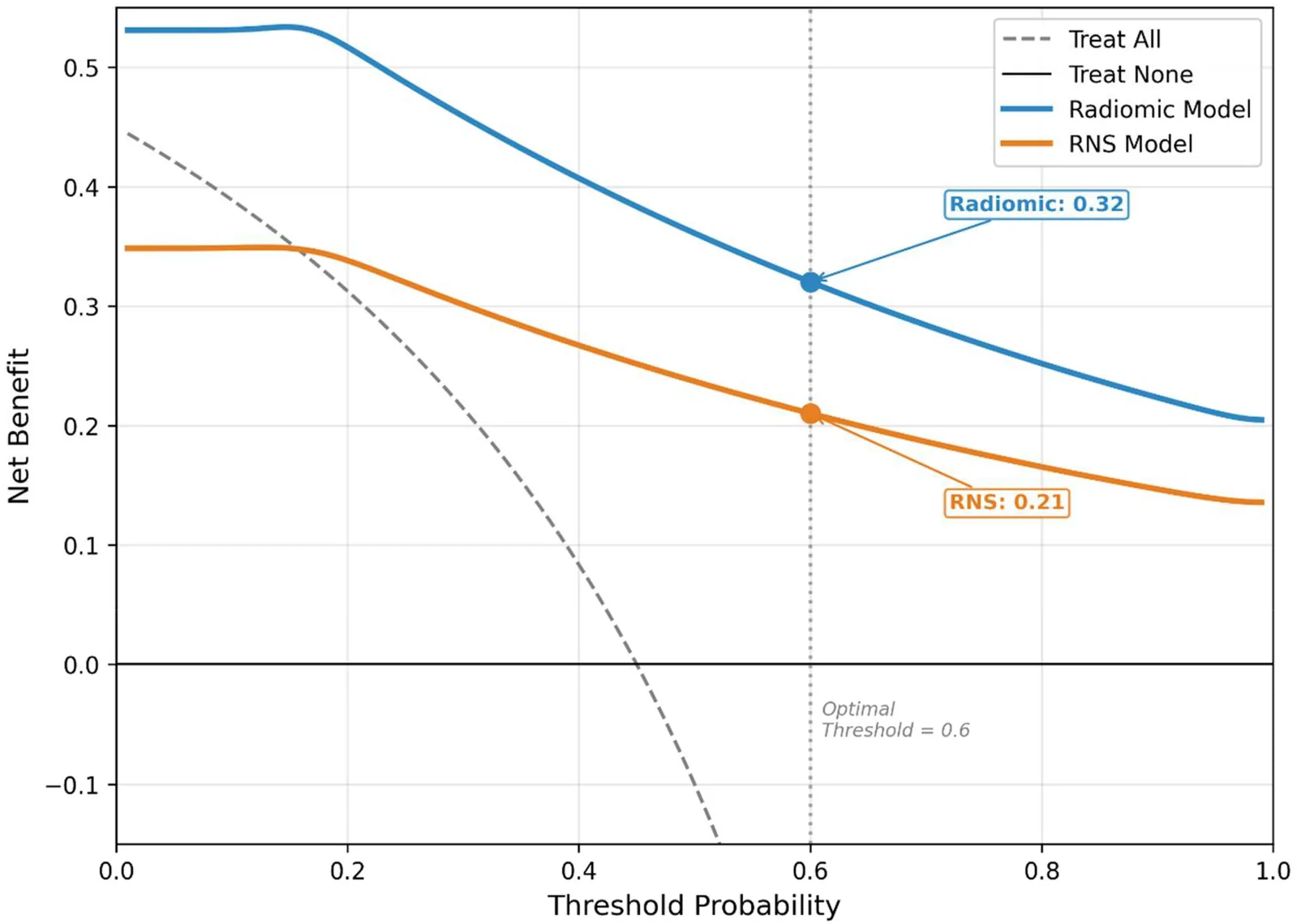
Decision curve analysis for the RNS model and Radiomic model.

SHAP analysis revealed that original_shape_Maximum2DDiameterSlice (tumor size) had the highest mean absolute SHAP value (0.32), confirming it as the most influential feature (**Figure 6**). Texture features, particularly log-sigma-2-0-mm-3D_glcm_Imc1 (mean absolute SHAP value = 0.28) and log-sigma-2-0-mm-3D_ngtdm_Strength (mean absolute SHAP value = 0.21), were also key contributors. Lower values of log-sigma-2-0-mm-3D_glcm_Imc1 (indicating less texture complexity) were associated with higher PN likelihood. This feature quantifies the complexity of gray-level co-occurrence patterns, with higher values indicating more complex, less predictable texture. In ccRCC, increased texture complexity has been associated with higher Fuhrman grade, greater microvascular density, and increased intratumoral heterogeneity (16, 27). Tumors with greater heterogeneity may exhibit more infiltrative growth patterns, less distinct pseudocapsules, or more necrotic areas, all of which could complicate surgical resection.

**Figure 6:**
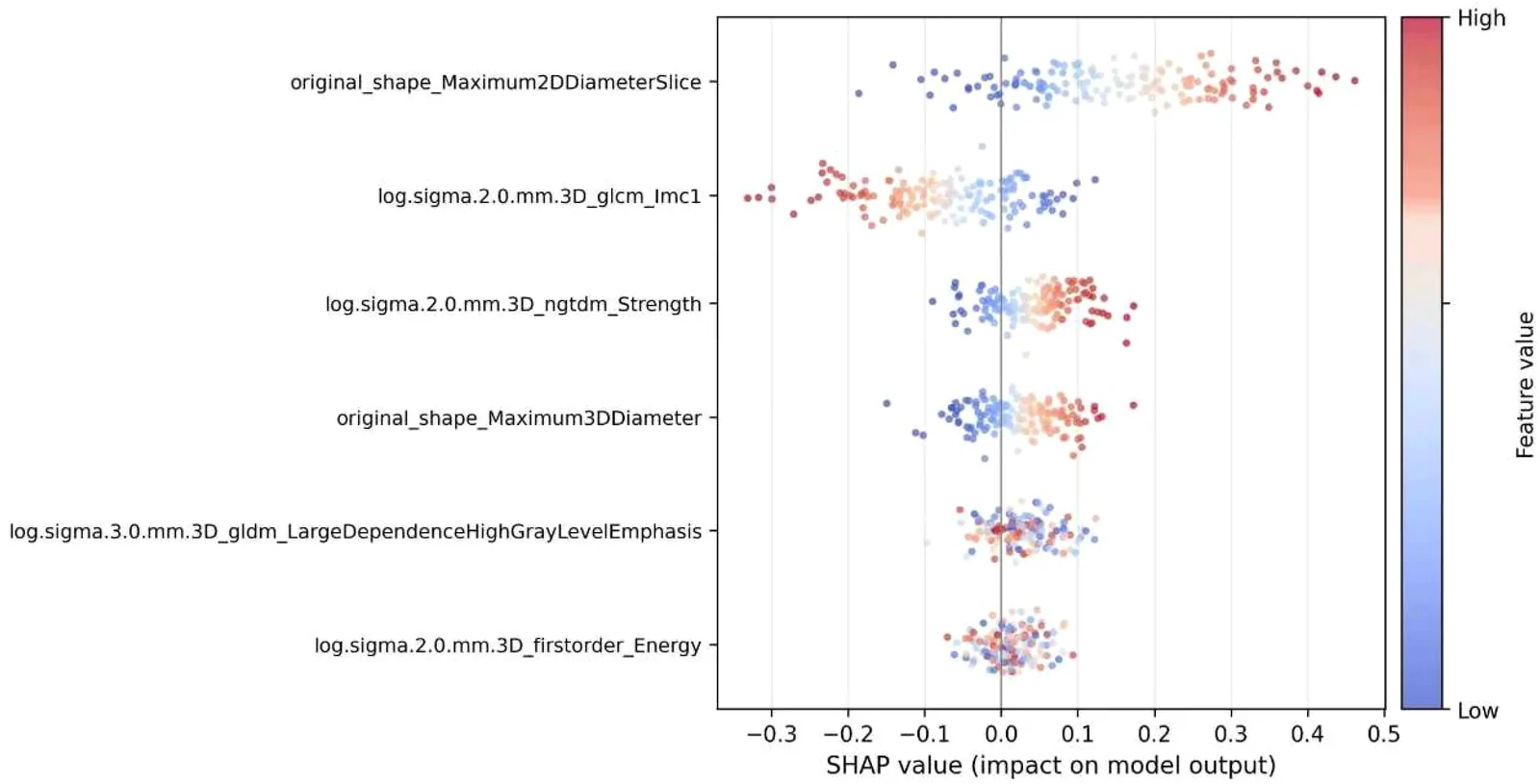
SHAP (SHapley Additive exPlanations) feature importance analysis.

## Discussion

This study demonstrates that, while the established RNS is a valuable tool for describing renal tumor anatomy and correlates with surgical approach, a CT-based radiomic model provides a more accurate prediction of the likelihood of undergoing PN in patients with ccRCC. Key findings include: 1) the radiomic model significantly outperforms the RNS model (AUC 0.886 vs. 0.793); 2) feature reproducibility analysis (ICC > 0.75) supports the robustness of our approach; 3) SHAP analysis identifies tumor size and texture heterogeneity features as key predictors; and 4) the radiomic model demonstrates better calibration and clinical utility.

Our findings align with the core principle of the RNS: larger size (R), endophytic nature (E), and central location (L) increase anatomical complexity, making PN more challenging (5, 6). However, the superior performance of the radiomic model suggests that it may capture quantitative image features—including texture patterns reflecting intratumoral heterogeneity—that are not fully captured by conventional size and shape measurements (28, 29). This likely reflects differences in underlying tumor characteristics, such as fibrous capsular reaction, microvascular patterns, or necrosis, which affect the technical ease of dissection and reconstruction during PN (30, 31). The finding that larger maximum 2D diameter was associated with higher predicted probability of PN warrants careful interpretation. This likely reflects the fact that in our cohort, surgeons were more willing to attempt PN for larger but anatomically favorable tumors (e.g., exophytic, peripheral lesions), whereas small endophytic or centrally located tumors—which are technically challenging despite their small size—were more likely to undergo RN. Thus, the association between size and PN likelihood is context-dependent and should not be interpreted as a general principle that ‘larger tumors are easier to remove.

It is essential to emphasize that the model predicts the observed surgical approach (the treatment actually received) rather than an objective measure of tumor resectability or the ‘optimal’ procedure. This distinction is fundamental to interpreting our findings: the model’s performance reflects how well radiomic features correlate with real-world clinical decision-making, which incorporates surgeon judgment, patient preferences, and institutional practices, rather than purely anatomical suitability for PN.

An important methodological consideration is that RNS was one of several inputs to the actual surgical decision-making process, yet we compare the RNS model against the radiomic model as if they were independent predictors. This creates a degree of ‘circularity’ in the RNS comparison: the RNS model’s performance reflects how well a scoring system—which was part of the decision process—predicts the outcome it helped inform. This likely biases the RNS model’s performance upward (since it was correlated with the decision) rather than downward, meaning the radiomic model’s superior performance is particularly noteworthy as it outperforms a score that was already influencing clinical decisions.

Additionally, Fuhrman grade differed significantly between PN and RN groups (65.7% vs. 37.6% low-grade, P < 0.001) and correlated with both tumor morphology and, plausibly, surgical decision-making. However, grade is not known preoperatively and therefore was not included as a predictor in our models, consistent with our goal of developing a purely preoperative tool. We did perform a *post-hoc* sensitivity analysis adjusting for Fuhrman grade (**Supplementary Table 2**), which showed that the radiomic model’s performance remained significant (AUC = 0.875, 95% CI: 0.80-0.91) after grade adjustment, suggesting that the radiomic signal is not merely a surrogate for tumor grade.

The non-monotonic relationship between RNS complexity and RN rate—with the highest RN rate observed in the moderate-complexity group rather than the high-complexity group—merits further discussion. This pattern may reflect selection bias, small sample size in the high-complexity group, and institutional practice patterns. These findings underscore that RNS complexity does not translate linearly into surgical approach in real-world practice, highlighting the need for more nuanced predictive tools.

Our radiomic model performance (AUC = 0.886) is numerically comparable to recent studies (16, 17, 32), though direct comparisons should be interpreted with caution given the substantial difference in endpoints (surgical decision vs. pathological grade). To our knowledge, this study is among the first to directly compare radiomics with RNS for predicting actual surgical decisions.

Based on DCA, a potential clinical implementation framework can be hypothesized, but must be considered preliminary given the single-center, internally validated nature of our model. At a threshold probability around 0.6, the radiomic model showed favorable net benefit in our cohort. However, this threshold should not be adopted clinically without external validation in independent cohorts from centers with different practice patterns. We present this framework as a proof-of-concept illustration rather than a ready-to-use clinical decision rule.

Despite our efforts to ensure methodological rigor, several limitations of this study warrant discussion. First, because we predicted the actual surgical approach (PN vs. RN) rather than an objective measure of resectability, our endpoint is inherently tied to local practice patterns, surgeon expertise, and patient referral pathways at our single institution. Consequently, the model’s performance may not generalize to centers with different thresholds for attempting PN. External validation across diverse clinical contexts—ideally with calibration to local PN rates—is essential to distinguish whether our predictions reflect universal biological principles or institution-specific biases. Second, our cohort was restricted to patients with pathologically confirmed ccRCC, yet this histology is rarely known preoperatively. Therefore, the current model serves as a proof-of-concept for ccRCC, and its utility as a preoperative decision-support tool for unselected renal masses must be prospectively validated using imaging-based presumptive diagnoses. Methodologically, while we rigorously assessed radiomic feature reproducibility with ICC > 0.75, the RNS was evaluated by a single reader, and we acknowledge that its interobserver variability was not independently quantified for this comparator. Additionally, manual tumor segmentation remains time-consuming and inherently variable, highlighting the potential for automated deep-learning segmentation to improve efficiency in future iterations. Furthermore, despite standardized preprocessing, radiomic features remain sensitive to scanner manufacturers and acquisition protocols; batch effects cannot be fully eliminated, reinforcing the need for multicenter validation with diverse scanning protocols. Finally, we hypothesized that the selected texture features reflect underlying tumor biology, but we did not perform direct histopathological correlation—such as matching specific radiomic signatures to nuclear grade, necrosis, or fibrosis. Integrating such molecular or pathological data would strengthen the biological underpinnings of the model. Ultimately, the true test of any prognostic tool lies in its ability to predict patient outcomes. Thus, future studies should incorporate long-term follow-up data to examine whether radiomic predictions of surgical approach correlate with overall survival and progression-free survival.

## Conclusion

The RNS is a useful tool for describing renal tumor anatomy. However, for predicting the likelihood of undergoing nephron-sparing surgery in clinical practice, a CT-based radiomic model derived from standard preoperative scans offers superior, objective accuracy. This data-driven approach captures both anatomical and quantitative tissue characteristics relevant to surgical decision-making. With rigorous external validation, it has the potential to become a valuable decision-support tool, assisting clinicians and patients in selecting the most appropriate surgical strategy for RCC. Prospective multi-center validation is warranted before routine clinical adoption.

## Data Availability

The raw data supporting the conclusions of this article will be made available by the authors, without undue reservation.
