# Supplementary material for: Radiomic model versus R.E.N.A.L. nephrometry score for predicting the likelihood of undergoing partial nephrectomy in clear cell renal cell carcinoma
Source: Front Urol. 2026 Sep 18;6:1928982. doi: 10.3389/fruro.2026.1928982 (PMC13630628; doi:10.3389/fruro.2026.1928982)
Supplement: Supplementary file 1 [file DataSheet1.pdf]

Supplementary Table S1 (Baseline Balance of Imperative Indications)

Characteristic	PN Group (n=312)	RN Group (n=101)	P-value*
Solitary kidney	5 (1.6%)	3 (3.0%)	> 0.05
Bilateral or multifocal tumors	9 (2.9%)	3 (3.0%)	> 0.05
Hereditary syndromes	2 (0.6%)	1 (1.0%)	> 0.05
Preoperative renal dysfunction (eGFR < 60 mL/min/1.73m²)	22 (7.1%)	9 (8.9%)	> 0.05

Note: Data are presented as n (%). P-values indicate no statistically significant difference between th e two groups, demonstrating that the distribution of imperative indications was balanced.

Supplementary Table S2 (Sensitivity Analysis Adjusting for Fuhrman Grade)

Model	Adjusted Variable	AUC (95% CI)
Primary Model (Radiomic Model)	None (Unadjusted)	0.886 (0.81 – 0.91)
Sensitivity Analysis Model	Adjusted for Fuhrman grade	0.875 (0.80 – 0.91

Note: After adjusting for postoperative pathological Fuhrman grade (which is unavailable preoperativ ely in routine practice), the radiomic model maintained a strong AUC of 0.875. This suggests that t he radiomic signal provides independent predictive value beyond simply being a surrogate for tumo r grade.

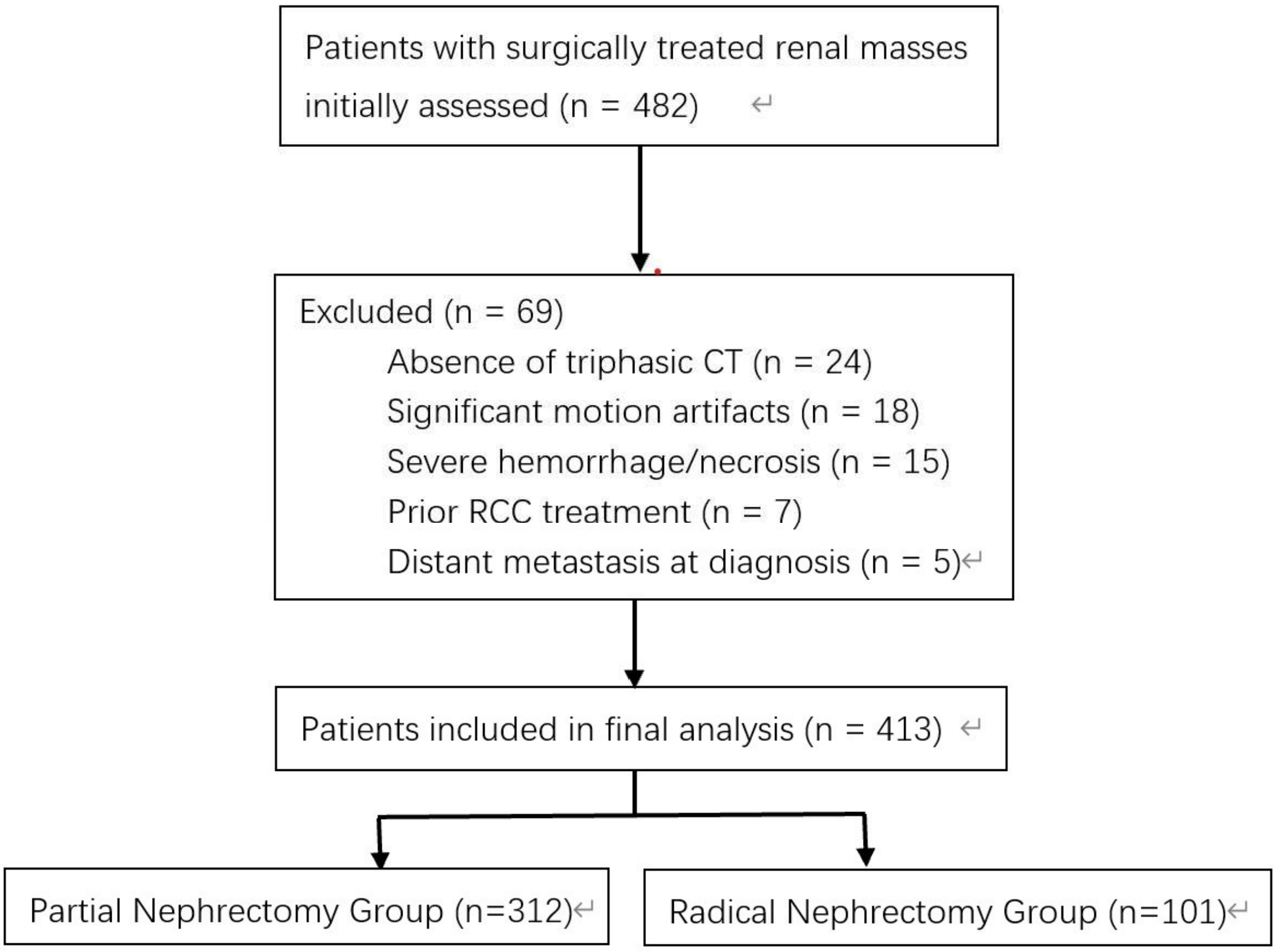

Supplementary Figure S1 ： Patient Selection Flowchart
